# Supported self-management for adults with type 2 diabetes and a learning disability (OK-Diabetes): study protocol for a randomised controlled feasibility trial

**DOI:** 10.1186/s13063-015-0832-9

**Published:** 2015-08-08

**Authors:** Rebecca E. A. Walwyn, Amy M. Russell, Louise D. Bryant, Amanda J. Farrin, Alexandra M. Wright-Hughes, Elizabeth H. Graham, Claire Hulme, John L. O’Dwyer, Gary J. Latchford, Alison J. Stansfield, Dinesh Nagi, Ramzi A. Ajjan, Allan O. House

**Affiliations:** Clinical Trials Research Unit, University of Leeds, Leeds, UK; Leeds Institute of Health Sciences, University of Leeds, Leeds, UK; Leeds and York Partnership NHS Foundation Trust, Leeds, UK; Mid Yorkshire Hospitals NHS Trust, Wakefield, UK; Division of Cardiovascular and Diabetes Research, University of Leeds, Leeds, UK

**Keywords:** Randomised controlled trial, Self-management, Learning disability, Type 2 diabetes

## Abstract

**Background:**

Individuals with a learning disability (LD) are at higher risk of developing type 2 diabetes, but LD is not straightforward to define or identify, especially at the milder end of the spectrum, which makes case finding difficult. While supported self-management of health problems is now established, current material is largely educational and didactic with little that facilitates behavioural change. The interaction between the person with diabetes and others supporting their care is also largely unknown. For these reasons, there is considerable work needed to prepare for a definitive trial. The aim of this paper is to publish the abridged protocol of this preparatory work.

**Methods/Design:**

Phase I is a prospective case-finding study (target n = 120 to 350) to identify and characterise potential participants, while developing a standardised supported self-management intervention. Phase II is a randomised feasibility trial (target n = 80) with blinded outcome assessment. Patients identified in Phase I will be interviewed and consented prior to being randomised to (1) standard treatment, or (2) supported self-management. Both arms will also be provided with an ‘easy read’ accessible information resource on managing type 2 diabetes. The intervention will be standardised but delivered flexibly depending on patient need, including components for the participant, a supporter, and shared activities. Outcomes will be (i) robust estimates of eligibility, consent and recruitment rates with refined recruitment procedures; (ii) characterisation of the eligible population; (iii) a standardised intervention with associated written materials, (iv) adherence and negative outcomes measures; (v) preliminary estimates of adherence, acceptability, follow-up and missing data rates, along with refined procedures; and (vi) description of standard treatment.

**Discussion:**

Our study will provide important information on the nature of type 2 diabetes in adults with LD living in the community, on the challenges of identifying those with milder LD, and on the possibilities of evaluating a standardised intervention to improve self-management in this population.

**Trial registration:**

Current Controlled Trials ISRCTN41897033 (registered 21 January 2013).

## Background

### Definition and case finding

The prevalence of type 2 diabetes varies markedly by ethnicity and social factors including deprivation. Case finding for service planning and for research is greatly facilitated in the UK by a requirement for general practitioners (GPs) to maintain a register of all patients with diabetes, remunerated through the Quality Outcomes Framework (QOF) for undertaking various health assessments on an annual cycle (http://cks.nice.org.uk/diabetes-type-2). Currently in the UK, approximately 5 % of the adult population is recorded on these diabetes registers.

Individuals with learning disability (LD) are at particular risk of developing type 2 diabetes [1], but the effectiveness of self-management in this group remains unclear. Identifying those with LD and diabetes would facilitate an understanding of the size of the problem in order to allow the implementation of effective management strategies. This is a complex area because LD is not straightforward to define and identify, especially at the milder end of the spectrum. It can be defined statistically based on test scores that typically show a negatively skewed distribution. In those terms, it is often said that 2 % of the general population will have some degree of LD (http://www.learningdisabilities.org.uk/help-information/Learning-Disability-Statistics-/) [2, 3]. However, the picture becomes more complex when an element of functional impairment in real-world activities is built into the definition, partly because any functional deficit may not be entirely attributable to intellectual impairment but to emotional or social problems. Conversely, an adult with intellectual impairment may not come to the attention of statutory or non-statutory agencies if he or she is functioning independently or is well supported by family or some other informal carer. The functional approach is now widespread (http://www.rcn.org.uk/__data/assets/pdf_file/0006/78765/003184.pdf; http://www.bps.org.uk/). It is estimated that a minority (≤25 %) of the adult population with LD is known to health or social services [4]. This is unfortunate because it is apparent that adults with LD have high rates of physical illness, and a recent report highlighted their poor levels of healthcare [2, 5].

Supported self-management of long-term conditions such as diabetes is now quite well established, although its content and the intensity with which it is delivered, have varied considerably between studies [6]. Current self-management materials largely support information provision with less emphasis on facilitating behavioural change. Moreover, many adults with LD do not live independently even when they can be defined as living outside hospital or residential care. This means self-management must be negotiated not just with the person with diabetes but with their supporter; therefore, considerable flexibility is needed to negotiate and implement an intervention. For these reasons, there is considerable preparatory work to do before a definitive trial can be designed and even before a feasibility trial can be undertaken. This paper describes the protocol for the preparatory work for a definitive trial undertaken within the OK Diabetes study.

### Aims and objectives

The aims of Phase I of this study are as follows:

1. To develop and evaluate a method to identify participants who have mild/moderate LD *and* type 2 diabetes who are not taking insulin and who might be suitable for supported self-management, including procedures for establishing capacity and consent, and to characterise this population.
2. To develop a standardised but flexible intervention supported by written materials to aid supported self-management of diabetes, a simple measure of adherence to the intervention, and to assess the feasibility of delivering the intervention.

The aims of Phase II of this study are to undertake a feasibility trial:

1. To assess the feasibility of (i) collecting and recording a range of physiological, psychological, behavioural and cost-effectiveness outcome measures, (ii) maintaining the blind for subjective outcomes, (iii) collecting data from medical records and (iv) using the adherence measure to assess delivery and use of self-management techniques.
2. To develop a checklist of potential negative outcomes and a related process for their collection, with a preliminary assessment of the acceptability of the intervention (via adherence, drop-outs and negative outcomes such as distress and agitation) and to provide a detailed description of what treatment is delivered to each arm.
3. To estimate parameters needed for the design of a definitive Phase III trial (for example, recruitment, adherence and retention rates, and variability in HbA1c, BP, BMI, and EQ-5D).

## Methods/Design

### Design

Phase I is a prospective case-finding survey involving 120 to 350 participants. Phase II is an individually-randomised controlled feasibility trial of supported self-management plus standard treatment versus standard treatment, involving 80 participants (and where possible their supporters). Eligible, consenting participants will be randomised on a 1:1 basis and followed up for 6 months. Identified supporters will also be consented to the trial. Participants, supporters, referrers and care providers cannot be blinded to treatment allocation. Steps will therefore be made to blind all aspects of outcome assessment carried out by researchers. Data will be collected from medical notes, by the nurse and by researcher interview. Where appropriate, data will be used to inform whether it is feasible to design a definitive, multicentre RCT.

### Setting

Three districts in West Yorkshire, centred in the cities of Leeds, Bradford and Wakefield, were selected to take part to maximise generalisability and provide a good test of feasibility of recruitment. Service configurations vary considerably across these three settings. In Leeds, most type 2 diabetes cases are managed exclusively in primary care, with referral to secondary care only for specific problems. There is a tiered system for diabetes care in Bradford, with almost half of the GP practices managing insulin transfer, and several participating in collaborative care of complex cases. In Wakefield, primary care diabetes services are supported by local commissioning arrangements, where specialist teams tailor their work to individual need. There are also a wide range of services for people with LD in the social care and third sectors.

### Eligibility criteria

**Fig. 1:**
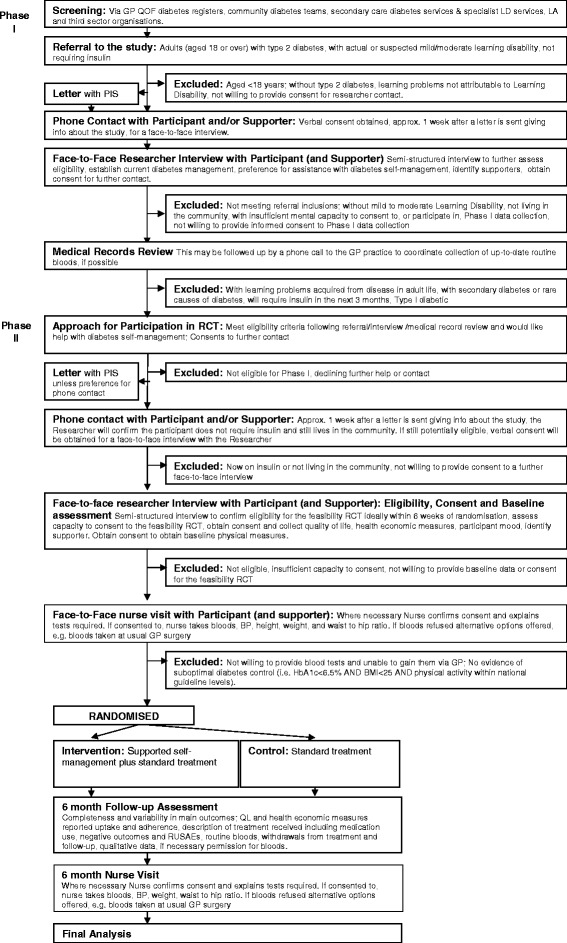
Flow Diagram

Participant eligibility will be assessed in stages (see Fig. 1 for details). Although we intend to recruit people with LD and type 2 diabetes even if they live independently and have no informal supporter, we expect the majority will have a supporter.

#### Inclusion criteria

The inclusion criteria are as follows:

1. Aged 18 years or over (Phase I and II)
2. Diagnosed with type 2 diabetes, controlled with diet alone or hypoglycemic agents other than insulin (Phase I and II)
3. Mild to moderate LD (Phase I and II)
4. Living in the community (not in a hospital setting) (Phase I and II)
5. Up-to-date (ideally within 6 weeks of randomisation, no maximum explicitly set) values of HbA1c and BMI (Phase II)
6. Suboptimal diabetes control, defined as HbA1c>6.5 % (equivalent to 48 mmol/mol) OR BMI>25 kg/m^2^ OR physical activity below national guidelines (Phase II)

#### Exclusion criteria

The exclusion criteria are as follows:

1. Insufficient mental capacity to consent or to participate in the research (Phase I and II)
2. LD acquired from disease in adult life, defined as 16 years or over, such as LD due to adult-onset dementia or stroke (Phase I and II)
3. Type 1 diabetes, secondary diabetes (such as steroids, pancreatitis, endocrine disorders *etcetera*) and rare causes of monogenic diabetes (such as maturity onset diabetes of the young) (Phase I and II)
4. Referred for insulin (Phase I) or put on insulin between identification and randomisation, or likely to require insulin in the next 6 months (Phase II)
5. Declining further assistance with diabetes self-management (Phase II)

A supporter will be defined as the main adult nominated by the supporter, by the participant or by a professional who knows that person, as providing practical help and support in day-to-day living relevant to their diabetes management. For Phase II an operational definition is ‘a key person in providing regular practical support in diabetes self-management, who is in contact with the person with diabetes at least weekly’ and able to give informed consent. It is recognised that there may be several people involved with relevant aspects of a participant’s life. They may be included as “other helpers” in self-management plans if desired by the participants but will not be defined as a supporter.

Mild to moderate LD will be defined by a researcher-led assessment of functional deficits (in daily activities, educational and social attainment and support needs, day-to-day cognitive functions of memory, knowledge) attributable to primary or secondary/acquired cognitive impairment. Mental capacity will be assessed by the researcher, trained by the LD consultant, following guidelines from the 2005 Mental Capacity Act (http://www.legislation.gov.uk/ukpga/2005/9/contents). Careful note will be made of grounds for inclusion or exclusion in each case using journaling techniques, recognising that a challenge will be to identify people with mild LD who are not already so-designated by themselves or others and yet who might benefit from the intervention.

### Participant identification and recruitment

All patients on registers in primary and secondary care will be screened for referral by staff in a range of settings - including GPs, primary care staff conducting QOF diabetes or LD checks or in community diabetes teams, secondary care staff in diabetes or specialist LD services, Local Authority LD services, or third sector organisations. In addition to screening QOF registers, general practices will be provided with an electronic (that is, Read Code) search, created by the research team, which identifies potentially eligible participants from electronic GP records, the results of which clinical staff will review. Once identified, referrers will obtain and document consent from the potential participant to pass on their contact details (or those of a supporter if preferred) to the research team. A letter and information sheet will then be sent from the research team to the potential participant (and/or supporter if identified), followed up a week later by a telephone call where the study can be discussed further. During the call, verbal consent will be obtained for a face-to-face interview with the researcher.

The first interview will use a standardised semi-structured interview format designed to (i) discuss the information sheet and obtain written or verbal (where necessary) informed consent for Phase I, (ii) obtain consent to review medical records for routine clinical measures, (iii) establish diabetes management, including diet and physical activity, (iv) elicit preferences for further assistance and consent to re-contact for Phase II, and (v) identify the role of supporters in diabetes management, nominating a supporter to be involved in Phase II as applicable. Local sites or the research team will provide an interpreter if either (or both) the participant or (and) supporter is (are) not fluent in English. In the rare event that no-one suitable is available, this will act as an exclusion criterion. During the Phase II baseline interview, the researcher will (i) further confirm eligibility (excluding suboptimal diabetes control where blood results are not yet available), (ii) seek consent to participation in Phase II, (iii) collect baseline data and, as appropriate, (iv) consent for a nurse to visit and take bloods and other physical measures prior to randomisation. If present and willing, a supporter will also be consented into the study. The researcher will send participants a certificate of participation after the 6-month follow-up.

### Registration and randomisation

Participants will be entered into Phase I at registration and Phase II at randomisation. Consent is obtained prior to registration and randomisation. All referrals that show documented consent will be registered, using a secure, automated 24-hour telephone registration service based at the Clinical Trials Research Unit (CTRU), University of Leeds. Following registration, researchers will follow up with participants’ GPs to determine final eligibility for Phase I via medical notes. If a participant meets the eligibility criteria for Phase II, they will be randomised, following informed consent and baseline assessment, on a 1:1 basis to receive supported self-management plus standard treatment or standard treatment using a secure, automated 24-hour telephone randomisation service based at the CTRU, ensuring allocation concealment. A computer-generated minimisation algorithm incorporating a random element will be used, accounting for (i) site (Leeds, Bradford, Wakefield), (ii) supporter (none, not living with supporter, living with supporter), (iii) HbA1c (≤48, 49 to 69 years of age, ≥70 mmol/mol), (iv) BMI (≤25 or >25 kg/m^2^), and (v) physical activity (below, or at or above national guidelines). The CTRU will inform the nurse, but not the researcher, of the outcome of randomisation. The nurse will contact participants, supporters and advocates as appropriate in the intervention and control arm to explain the outcome and to make an appointment with those in the intervention arm.

### Interventions

At the start of the study, we expected much of the work with people with LD to be under-taken through the involvement of informal supporters who are already providing input and whom we would not wish to by-pass or displace. This is in contrast to the usual pattern of support, where a professional or trained peer acts as a therapist to help and encourage the nominated patient in using the self-management materials. We will modify the format of materials, creating more visual, accessible, ‘easy read’ materials more appropriate to those with literacy problems. The developed interventions are briefly described below.

#### Supported self-management

Professional support will be arranged via a diabetes nurse. Two dedicated research nurses will provide support to participants across sites. Supervision and training will be given to the nurses by the intervention developers. The intervention has four standardised components with associated materials; how they are delivered will depend upon participant and supporter characteristics and preferences:

1. Establishing the participant’s daily routines and lifestyle: This includes current diet and activity routines, participation on daytime social activities or work, shopping and food preparation, current self-reported health and self-management.
2. Identifying all supporters and helpers and their roles: A key supporter will be identified where possible, and their role in the life of the person with diabetes. Key supporters and other helpers will be given written information about the project and if they agree to support a goal set by the participant they will be given a written reminder of their role.
3. Setting realistic goals for change: The main aim is to avoid prescribing change in the way of good dietary practice or other lifestyle change, but to support goals suggested by the person with diabetes that are specific, simple and achievable given the person’s current routines and social support, and consonant with their willingness to make change.
4. Monitoring progress against agreed upon goals: We have devised a simple system that does not depend on high levels of functional literacy. The outputs will be collected by the nurse delivering the intervention.

The nurse will work through the elements of supported self-management with the participant, explaining how to use materials and suggesting initial actions and activities. Further contact will be negotiated, but we anticipate a total of three to four meetings of 30 to 60 minutes over 6 to 8 weeks, followed by telephone support and advice, with the balance offered to the person with diabetes decided by negotiation.

#### Standard treatment

All trial participants will receive standard treatment for their diabetes. Different staff and services will be involved in providing this care, as uncomplicated type 2 diabetes in the community is usually managed through primary care.

#### Both groups

To ensure participants have a standardised minimum level of care, we will provide standard information about type 2 diabetes, made accessible for people with LD, to all participants. This information will be selected from resources already produced by the NHS, by Diabetes UK or a LD charity and amended by the project team (with permission of the creators). It will cover key areas already identified as essential in diabetes self-management (that is, diet, exercise, medication use, and feet and eye care). The resource will be selected in collaboration with our Patient and Public Involvement (PPI) collaborators. We will not otherwise attempt to influence the content of standard treatment.

### Data collection

The following will be collected during Phase I (Table 1):

**Table 1:** Phase I summary of assessments

Assessment (including who is involved)				
	Referral	Phone	Face-to-face interview	Medical notes
Screening				
Demographic data including (i) age, (ii) gender, and (iii) ethnicity	X			
Preferred method of contact	X			
Eligibility and consent				
Eligibility (assessed by Referrer, Study Researcher; confirmed by Clinician), including reason for exclusion	X	X	X	X
Documented consent to contact (Ref, P^a^)	X			
Verbal consent to interview (P, S, R)		X		
Mental capacity to consent to Phase I (P, R)			X	
Written consent to Phase I (P, S, R), including access to GP notes for QOF			X	
Documented consent to re-contact (P, S, R)			X	
Baseline data (P, S, R)				
Presence and role of a supporter		X	X	
Demographic data including (i) Household composition, (ii) type of housing, (iii) marital status, (iv) nature of support, (v) employment status, and (vi) first language			X	
Current physical health state from QOF measures (HbA1c, BP, BMI, Q Risk)				X
Prescribed diabetes regime (medication)			X	X
Preferences for further assistance (P, S, R)			X	

BP=Blood pressure, BMI = body mass index, HbA1c = glycated haemoglobin

^a^ *Ref* = Referrer, *P* = Participant, *S* = Supporter, *R* = Researcher; QOF, Quality Outcomes Framework; GP, general practitioner

Phase II assessments will be undertaken at the following time points (see Table 2):

**Table 2:** Phase II baseline and follow-up assessments

	Med notes review/check	Pre- baseline phone call	Baseline interview	Nurse baseline	6 month medical/ nurse follow-up	6 month follow-up interview	Med notes follow-up
Eligibility and consent							
Presence and role of a supporter and/or research advocate			X			X	
Mental capacity to consent to Phase II (P, R)			X				
Eligibility for Phase II (assessed by Study Researcher)	X	X	X				
Consent for Phase II (P, S, R^a^)			X				
Follow-up data (P, S, R, N)							
Negative outcomes						X	
Related and Unexpected Serious Adverse Events						X	
Hospital attendances						X	
Current physical health state (for example, HbA1c, BP, BMI, weight, BP, HbA1C, cholesterol, HDL/ LDL triglycerides, Urea and Electrolytes, waist to hip ratio (N)	X			X	X		
Thyroid function, height (N)				X			
Q Risk, Retinal screening, Medication, Serum creatinine, micro-albuminuria							X
Details of Treatment Received						X	
Adherence to the intervention (N)						X	
Prescribed diabetes regime (diet, exercise)						X	
Questionnaires (completed at Researcher visit)							
Health Economics questionnaire (P and S interviewer administered) to cover health and social care costs, participant and supporter expenses and productivity costs			X			X	
Participant (P) mood (questions taken from Phase I)			X			X	
Health-related quality of life (EQ5D) (P)			X			X	

BP=Blood pressure, BMI = body mass index, HbA1c = glycated haemoglobin

^a^ *P,* Participant; *S,* Supporter; *R,* Researcher; *N,* Research Nurse

1. Medical notes review/check (prior to randomisation)
2. Pre-baseline phone call (prior to randomisation)
3. Baseline interview (prior to randomisation)
4. Nurse visit (where necessary, prior to randomisation)
5. 6-month follow-up interview and qualitative interview
6. 6-month nurse/medical follow-up

with a guidance window of plus or minus 2 weeks at follow-up (baseline interviews should take place no more than 6 weeks prior to randomisation). If up-to-date Phase I HbA1c and BMI are available from their GP (available within the last 6 weeks) as established by the researcher, records may be carried forward to the Phase II baseline.

Participants, and their supporters, will be followed up directly by the researcher and, where appropriate, at separate visits by the nurse to collect blood and physical measures, at 6-months post-randomisation. Following the 6-month follow-up assessments, we will un-blind the researchers so they can interview participants in the intervention arm using a simple topic guide, followed by framework analysis to identify themes related to both positive and negative experiences of implementing the intervention. All participants who enter the study will be considered part of the intention-to-treat population, and efforts will be made to follow them up wherever appropriate. If there are no up-to-date medical records, then the nurse will collect weight, BP, waist-to-hip ratio (using a standard protocol), HbA1C, cholesterol, HDL/LDL triglycerides, urea and electrolytes. Wherever possible, the nurse visit will be undertaken blind.

### Health economics

In Phase I, we will develop data-collection forms to assess the cost effectiveness of the self-management intervention in a definitive trial, field testing them with service users from our PPI collaborators. It is generally argued that a societal perspective is adopted in economic evaluation of self-management interventions, as patients’ costs are likely to be more important than with more conventional interventions [7]. Thus, drawing on existing literature in LD and diabetes, the forms will identify patient resource use from the perspective of the health and social care sector and from a wider societal perspective. They will include the costs of health and social care (service provision and use of other health and social care services) and take into account the productivity costs (time away from work) and out-of-pocket expenditures incurred by the patients (for example, travel expenses, over-the-counter medicines and supplements and additional costs/savings of any dietary changes). Burden to the supporter will also be considered (for example, productivity costs and out-of-pocket expenses). In addition to participant/supporter-completed data, the resources associated with development and delivery of the intervention will be recorded. These will be based on routine data such as administrative records and participant records, as well as a detailed description of the development process. For both sets of data, unit costs for health service resources will be obtained from national sources such as the PSSRU, the BNF and the NHS Reference cost database. If national unit costs are unavailable, finance departments of trusts participating in the study will be asked to provide local cost data. The mean of these costs will be used as the unit cost estimate in the analysis. In line with NICE recommended practice, any cost effectiveness analysis will require quality-adjusted life years (QALYs) and utility weights for each health state observed in a trial population. A 2001 HTA report that considered general health status measures for cognitively impaired populations found the EQ-5D to be superior compared with other preference-based measures of health [8]. We will explore use of the EQ-5D for participants and for supporters/informal carers (not including ‘paid’ supporters) and of obtaining details of resources used from GPs.

### Outcomes

At the end of Phase I, we will: (i) have a robust estimate of the number of people who meet our eligibility criteria, and the number willing to consider change and to participate in further research, (ii) be able to characterise the population in terms of important characteristics such as diabetes control, living circumstances and role of supporters in diabetes management, (iii) have developed our intervention materials and adherence measures and field-tested them for acceptability in service users from our PPI collaborators, and iv) identified existing information resources on diabetes self-management suitable for people in our target group. Phase II will inform the choice of primary outcome for the definitive trial. The outcomes listed below relate to feasibility of recruitment, retention, intervention delivery and outcome collection.

- Recruitment and retention: (i) number of Phase I participants screened for eligibility for Phase II; (ii) proportion of Phase I participants screened found to be eligible for Phase II; (iii) proportion of Phase I participants that consent to Phase II out of those found eligible; (iv) proportion of Phase I participants randomised out of those that consent (prior to medical notes review); (v) proportion of randomised participants that have a supporter; (vi) reasons for non-participation (participant and supporter); (vii) method of identification for randomised participants and supporters; and (viii) proportion of randomised Phase II participants with all the required baseline and follow-up assessments completed, number of physical measures refused, number of withdrawals from follow-up data collection, reasons for withdrawal, number of losses to follow-up.
- Intervention delivery: (i) proportion of participants randomised to intervention attending at least one intervention session with the nurse, (ii) number of drop-outs from the intervention, reasons for drop outs, (iii) agreed upon method of measuring participant, supporter and nurse adherence to the intervention, including uptake/adherence rates and assessment of the feasibility of using a standardised measure, (iv) detailed description of what treatment was delivered to and received by each arm, including a comparison of standard treatment across arms plus an assessment of feasibility of collecting data on standard treatment pathways, and (v) preliminary assessment of the acceptability of the intervention, including negative outcomes, hospital attendances and RUSAEs.
- Outcome data collection: (i) assessment of the feasibility of blinding researchers and follow-up nurse to treatment allocation, (ii) proportion of participants who refuse physical measures with available and timely QOF data at baseline and (if necessary) at their follow-up assessments, (iii) assessment of the feasibility of collecting data on adherence to the intervention and the standard information, (iv) completion rates for other data collected, including assessment of the feasibility of collecting health economics data (for example, participant EQ-5D, NHS and supporter costs, medication use), and (v) missing item level data on self-reported questionnaires as collected by the researcher.
- Statistical outcomes: (i) variability of candidate primary/secondary outcomes at 6 months post-randomisation (HbA1c, BP, BMI, waist to hip ratio, EQ-5D, vascular/micro-vascular risk markers, participant mood), (ii) proportion of participants classed as abnormal on standard criteria for medical markers, and (iii) assessment of the potential for contamination within households.
- Qualitative outcomes: (i) positive and negative experiences of supported self-management, (ii) perceptions of standard information resource and experience of being in control arm, (iii) further detail on standard treatment in all participants, and (iv) modified implementation plan for the definitive trial.

### Monitoring adherence

Steps taken to ensure consistency in the use of the intervention will be recorded (training and supervision sessions with nurses, annotation of the manual by nurses, other experience and training in diabetes or LD care prior to Phase II). Provider adherence will be recorded by the nurse recording (i) dates of treatment meetings, (ii) all telephone contacts, (iii) materials provided (for example, goal-setting sheets, weekly planning and self-monitoring charts), (iv) confirmation that a named supporter (if there is one) has received an information pack. Participant and supporter adherence will be recorded by the nurse. Evidence of the use of techniques of self-management will be identified by copying and/or collecting: individual goal-setting charts; weekly planning charts; self-monitoring charts. These materials will be independently reviewed by a member of the research team, and data will be entered onto a form to detail use of each of the identified elements of the intervention. We will develop a coding and scoring system, with a global rating of overall adherence on a short ordinal scale. We will supplement quantitative data with qualitative interviews with all trial participants.

### Sample size

#### Phase I

We aim to interview up to 350 people meeting eligibility criteria, based on the assumption that there are 1,400 people across the three sites in the target population (2 % of the population with type 2 diabetes), with an anticipated 50 % GP involvement and 50 % eligible adults. This will allow us to estimate the proportion eligible of the target population (that is, conservatively 50 %) for Phase II to at least within 5.2 % with a two-sided 95 % confidence interval. If we recruit fewer participants, the width of this confidence interval will increase, reducing precision to 6.9 % if we recruit 200.

#### Phase II

To address the feasibility objectives, we plan to recruit 80 participants, randomised on a 1:1 basis, to obtain follow-up data on at least 30 participants per arm, as recommended by Lancaster *et al*. [9]. This assumes loss to follow-up will be no greater than 25 % at 6 months.

### Planned analyses

The statisticians are responsible for the statistical analyses for Phase I and II. The health economists are responsible for health economic analyses. A statistical analysis plan will be written and agreed before any analysis is undertaken, with any changes, with reasons, to the finalised plan documented.

#### Interim analyses

No interim analyses are planned.

#### Phase I analysis

To evaluate the case-finding method, the pattern/prevalence of uncertainty during eligibility assessment, method of participant identification, contacts including number, duration, and type, duplicate referrals and clusters of referrals will be summarised overall and by referrer role and region, where appropriate. To provide robust estimates of eligibility, screening, referral, interview, eligibility, capacity, consent and registration rates will be reported, with consent sub-divided into those willing to consider change and those willing to participate in further research. The eligible population will be characterised based upon the summary of diabetes control, demographics, living circumstances, and presence and involvement of a supporter in diabetes management. Candidate outcome measures of diabetes control available in routine care will be summarised in terms of data quality, and acceptability will be assessed by the presence of missing items.

#### Phase II analysis

Analysis will be conducted, when all the available data has been received and cleaned, on the intention-to-treat sample, including all randomised participants in the arm to which they were randomised. The flow of participants and supporters through the study will be depicted in CONSORT diagrams. Recruitment, uptake and adherence rates will be reported overall, by arm and by recruiting site. Six-month follow-up rates will be reported for each outcome (for participant and supporter, self-reported measures and those collected from routine GP data sources), overall, by arm and site. The candidate primary outcomes for a definitive RCT of HbA1c, BP and BMI will be summarised overall and by arm at baseline and 6 months and for the intervention arm by adherence to the manual at 6 months. Means and SDs, or medians and IQRs, will be presented, depending on the distribution, together with 95 % CIs. This will provide more accurate estimates of variability, recruitment, follow-up and adherence needed to assess the feasibility of recruiting into, and inform the sample size estimation of, a definitive trial. The feasibility and quality of collecting blood tests, vascular risk markers and markers of microvascular disease by the nurse will be reported and the proportion and nature of any missing data on these outcomes. The presence and quality of data collected on other outcomes will also be summarised, including adherence to the intervention at 6 months. This will help to inform the modes of data collection and the instruments used in a definitive trial. Outcomes of participant mood, negative outcomes and health-related quality of life, changes in treatment and patient and supporter/informal care will be reported with means/SDs, medians/IQRs or frequencies/proportions, depending on their distributions, together with 95 % CIs at baseline and 6 months both overall and by arm. The outcome measure relating to acceptability and uptake of the intervention will be assessed by the presence of missing items in the records of participants randomised to receive supported self-management.

#### Economic analysis

Whilst the primary aim of Phase II is to test the feasibility of data collection for any subsequent trial, analysis of the data collected will include descriptive statistics of the resources used. Within this Phase II analysis we will follow the NICE methods guidance [10] in as much as the perspective of the NHS and personal social services would be taken but additional analysis would adopt a societal perspective. A summary of the trial design is included as a flow diagram below (see Fig. 1).

### Ethical considerations

Ethical approval was granted for the study by the Yorkshire and Humber Research Ethics Committee (Reference: 12/YH/0304). We will include in both phases only those with the capacity to consent to the research, and in Phase II only those with the capacity to also undertake an element of self-management. We will also attempt to obtain consent from a supporter when that person supports diabetes self-management and is willing to be involved in the study. They will consent to assist the person with diabetes in any changes agreed during the intervention, and to participate as agreed in the project by, for example, assisting in collection of materials. Consent will be reassessed by the researcher and nurse at follow-up. The researcher will receive advice and support in this from third sector partners, and will be familiar with standard guidance on the topic [11]. If the participant agrees to participate then written informed consent will be obtained. Verbal consent will be obtained (where necessary) only when consent is clearly given but the participant cannot provide a signature or initials. Each consenting participant will be offered contact with a research advocate, who is independent of the research team. The advocate’s role is to provide independent support to participant’s regarding their participation throughout the trial and follow-up, including, for example a decision to withdraw from the research.

### Trial governance

The Project Management Group (PMG), comprising the Chief Investigator, project manager, co-investigators, members of 3^rd^ sector organisations (see below) and CTRU team will meet monthly and is responsible for the clinical set-up, on-going management and for the interpretation of results. The Trial Steering Committee (TSC), with independent Chair, will meet 6 monthly and provide overall supervision of the project, including trial progress, adherence to protocol, safety and consideration of new information.

### Public and patient involvement

We have worked closely, to develop this protocol, with two third sector organisations that represent the needs of people with LD in our region **-** supported by a grant from our local Research Design Service - People in Action (http://www.peopleinaction.org.uk/), and Tenfold (http://www.tenfold.org.uk/). During implementation of the project we will continue to work with both these organisations and with *easy on the i* (http://www.easyonthei.nhs.uk/), the information design service in the Learning Disability Service at Leeds and York Partnership NHS Foundation Trust.

## Discussion

We anticipate three main challenges in the delivery of this project. First, patient identification and recruitment may be difficult: although neither diabetes (prevalence 4–5 %) nor LD (prevalence 2 %) is very rare, in combination they have a prevalence of approximately 0.1 %. In a GP practice with an adult list size of 10,000 that means a pool of only 10 patients, from which there will also be exclusions due to patients having type 1 diabetes, or type 2 diabetes treated with insulin, insufficient mental capacity, not living independently in the community, or because they do not give consent. We will seek to maximise participation by developing and testing several methods of recruitment in primary care simultaneously, by seeking referral from community services (in dentistry, dietetics and podiatry for example) and secondary care and by active collaboration with 3^rd^ sector organisations.

A second challenge is that we know very little about how our target population manage type 2 diabetes or, as a group, what their diabetes control is like. This means that it is difficult to specify in detail the form and content of our intervention until we have recruited reasonable numbers into Phase 1, although the main components are clear as outlined above. Third is the choice of outcome measures. Our proposed primary outcome measure is HbA1c as it is the most common outcome in diabetes trials, and a cost-effective intervention will need to demonstrate an effect on this important marker of risk. However, other important determinants of longer-term outcomes in diabetes, such as diet and physical activity levels, are notoriously difficult to record reliably in the general population and are likely to be even harder in those with a LD. An important output of this study will be to establish what can be reliably and accurately collected in the way of secondary outcomes - including those related to the economic analysis.

## Trial status

Recruitment of participants is ongoing in three study districts in West Yorkshire, centred in the cities of Leeds, Bradford and Wakefield.

## Abbreviations

ADL: Activities of daily living
BMI: Body mass index
BNF: British National Formulation
BP: Blood pressure
CI: Chief Investigator or confidence interval
CRF: Case report form
CTRU: Clinical Trials Research Unit
EQ5D: EuroQol 5D
GCP: Good clinical practice
GP: General Practitioner
HbA1c: Glycated haemoglobin
HRQoL: Health-related quality of life
HTA: Health Technology Assessment
IQR: Inter-quartile range
LA: Local authorities
LD: Learning disability
LIHS: Leeds Institute of Health Sciences
MODY: Maturity Onset Diabetes of the Young
MRC: Medical Research Council
MREC: Multicentre Research Ethics Committee
NHS: National Health Service
NICE: National Institute of Clinical Excellence
NRES: National Research Ethics Service
PHQ9: 9-item Physical Health Questionnaire
PIS: patient information sheet
PMG: Project Management Group
PSSRU: Personal Social Services Research Unit
QALYs: quality adjusted life years
QL: Quality of Life
RCN: Royal College of Nursing
QOF: Quality Outcomes Framework
RCT: Randomised Controlled Trial
RUSAE: Related but Unexpected Serious Adverse Event
SAE: serious adverse event
SD: standard deviation
SDSCA: summary of diabetes self-care activities
SOP: standard operating procedure
TSC: Trial Steering Committee
